# Epigenetic changes reveal that vitamin B12 intake is associated with lower insulin resistance in older adults

**DOI:** 10.1186/s12263-026-00813-z

**Published:** 2026-08-04

**Authors:** Chao-Qiang Lai, Laurence D. Parnell, Yu-Chi Lee, Bruce S. Kristal, Oladimeji J. Akinlawon, Margaret P. Winderman, Tamara K. Reed, Liam Fouhy, Kelsey M. Mangano, Josiemer Mattei, Katherine L. Tucker, José M. Ordovás

**Affiliations:** 1 USDA ARS, Precision Nutrition Team, JM-US Department of Agriculture Human Nutrition Research Center on Aging at Tufts University, Boston, USA; 2 Precision Nutrition Team, JM-US Department of Agriculture Human Nutrition Research Center on Aging at Tufts University, Boston, USA; 3 Friedman School of Nutrition Science and Policy, Tufts University, Boston, USA; 4 Department of Biomedical and Nutritional Sciences, University of Massachusetts, Lowell, USA; 5 Department of Nutrition, Harvard School of Public Health, Boston, USA; 6 IMDEA Food Institute, CEI UAM + CSIC, Madrid, Spain; 7 Centro Nacional de Investigaciones Cardiovasculares (CNIC), Madrid, Spain

**Keywords:** Epigenetic changes, Epigenome-wide association study, Insulin resistance, Type 2 diabetes, Dietary intake, Vitamin B12, Sugar intake

## Abstract

**Background:**

Dietary choices influence both risk and development of type 2 diabetes (T2D), but the precise mechanisms remain incompletely described. Dietary intake and lifestyle behaviors can alter epigenetic states with subsequent modulations on gene expression which then can change the risk of T2D.

**Methods:**

An epigenome-wide association study was conducted to identify any epigenetic links to diabetes risk by investigating epigenetic changes during a 6-year follow-up in 374 participants who were free of T2D at baseline. The identified epigenetic changes were examined for their association with any dietary and lifestyle factors. Additionally, mediation analysis determined whether the associated dietary factors contribute to diabetes risk through epigenetic changes.

**Results:**

The epigenome-wide association study identified significant epigenetic changes during the 6-year follow-up at seven loci within genes *REPIN1*, *BMP2*, *LINC01723*, and *SEL1L2*, all of which were associated with insulin resistance. Further analysis revealed strong associations between three specific differentially methylated sites (DMSs) cg16400647 in *REPIN1*, cg12344389, and cg00625573, and dietary vitamin B12 intake. Additionally, cg26719314 on the X chromosome showed separate robust correlations with total sugar intake. Mediation analysis showed that higher vitamin B12 intake might reduce insulin resistance through epigenetic modification in *REPIN1* and *DNAAF9.* Conversely, higher total sugar intake correlated significantly with elevated diabetes risk, with cg26719314 acting as a mediator. Furthermore, increased vitamin B12 supplementation was associated with decreased insulin resistance at 6-year follow-up in the entire cohort.

**Conclusions:**

These findings suggest that vitamin B12 intake may influence insulin resistance through specific epigenetic changes, thus describing the epigenetic links between diet and development of T2D.

**Supplementary Information:**

The online version contains supplementary material available at https://doi.org/10.1186/s12263-026-00813-z.

## Introduction

Type 2 diabetes (T2D) poses a challenging and escalating public health burden, affecting a substantial portion of the global population [1, 2]. The prevalence of T2D is particularly pronounced with unhealthy dietary habits, such as high intake of ultra-processed foods, added sugar and refined carbohydrates [3], and within specific demographics in the USA, notably subpopulations struggling with obesity and the vulnerable population of older adults [4]. While a multitude of factors, encompassing genetic predisposition, environmental exposures, and lifestyle choices, contribute to the multifaceted landscape of T2D risk [5, 6], the precise molecular mechanisms inducing its pathogenesis remain elusive. The impact of diet and lifestyle on risk of T2D has been the focus of nutrition research for decades [7–12]. The effects of environmental exposures on the human genome are persistent, and habitual dietary intake and lifestyle behaviors can alter epigenetic states without changing the genomic DNA sequence. The altered epigenetic states can affect gene expression and the physiological functions of cells, cellular structures and organelles [13, 14]. These epigenetic modifications, including processes such as DNA methylation, histone modifications, and non-coding RNA regulation, are central mediators of gene-environment interaction [15–17]. In turn, changes in physiological functions can increase the risk of T2D [14]. However, the underlying mechanisms by which dietary and lifestyle exposures alter epigenetic states are not fully understood.

Among various epigenetic markers, DNA methylation, the addition of a methyl group, mostly to deoxycytosine but also to adenosine residues within DNA, has gained significant attention in the context of T2D [18–20]. Extensive research has demonstrated a strong association between specific DNA methylation patterns and the prevalence of T2D [21–23]. These methylation signatures can influence in certain tissues the expression of genes involved in key metabolic pathways, including insulin signaling [24, 25], glucose homeostasis [26], and lipid metabolism [27]. Consequently, aberrant DNA methylation profiles may contribute to the development of insulin resistance, a hallmark of T2D, and the subsequent progression to overt hyperglycemia.

Despite the growing body of evidence linking epigenetic modifications to T2D, the longitudinal dynamics of these changes and their predictive value for incident T2D in older adults remain undefined. To address this gap, this study aimed to investigate the longitudinal relationship between epigenetic changes, specifically DNA methylation, and the risk of developing T2D over a 6-year period in a well-characterized cohort of older American adults. By employing a prospective study design, we sought to elucidate the potential role of epigenetic mechanisms in the increase in insulin resistance as an indicator of T2D risk. Our primary objective was to identify specific DNA methylation sites that exhibit significant changes associated with the risk of type 2 diabetes and are linked to dietary intake, thus providing insight into the molecular pathways underlying this complex metabolic disease. The epigenetic link between dietary and lifestyle habits and disease risk could provide precise biomarkers and strategies for diabetes prevention within the framework of precision nutrition [28].

## Methods and materials

### Study population

#### Boston Puerto Rican Health Study (BPRHS)

The BPRHS is a longitudinal cohort study designed to identify interactions between stress, nutrition, and health outcomes (e.g., metabolic and cardiovascular disease) among Puerto Ricans living in the greater Boston area [29]. Interviews were conducted at three periods from 2004 to 2015: baseline, approximately 2 y after enrollment, and approximately 6 y after enrollment. Detailed methods for recruitment and data collection have been described [29, 30]. Comprehensive dietary, clinical, and biochemical assessments were performed on 1,311 of the 1,502 adult participants aged 45 to 75 y. A total of 958 completed the 6-year follow-up and, of these, 419 had been free of T2D at baseline. DNA samples were available for 374 at both baseline and 6-year follow up for Infinium HumanMethylation EPIC BeadChip v2 analysis. The flowchart of the study design is shown in Supplementary Fig. 1.

#### Type 2 diabetes and related traits

Blood samples were collected from participants after an overnight fast. Blood glucose was measured with a kinetic enzyme-linked immunosorbent assay (OSCR6121; Olympus America, Melville, New York, USA), and glycated hemoglobin (HbA1c) was determined with a latex immunoagglutination inhibition method on the Cobas FARA using the Roche Unimate HbA1c kit (Roche Diagnostics, Indianapolis, Indiana). Fasting serum insulin was assessed using an IMMULITE 1000 (Siemens Medical Solutions Diagnostics, Malvern, PA). T2D was defined as a fasting plasma glucose ≥ 126 mg/dL or self-reported use of glucose-lowering therapeutic agents. The HOMA-IR (homeostatic model assessment of insulin resistance) index was calculated based on the formula: fasting insulin (microU/L) x fasting glucose (mg/dL) x 0.055/22.5. HOMA-B (homeostatic model assessment of β-cell function) was estimated as 20 x fasting insulin (microU/ml)/[fasting glucose (mg/dl)/18 − 3.5] [31].

#### Dietary assessment

An adapted and validated food frequency questionnaire (FFQ) was used to collect dietary intake data in this population [32]. To include foods and portion sizes that represent a typical Puerto Rican diet, the FFQ was constructed based on the National Cancer Institute/Block FFQ and modified using dietary recalls from the Hispanic Health and Nutrition Examination Survey (HHANES) for the subset of Puerto Rican adults. Compared with the original Block questionnaire, the adapted FFQ was shown to be a better estimate of dietary intake in this Hispanic population [32]. Average daily nutrient intakes from both food and supplemental sources were determined using the Nutrient Data System for Research software, version 2007 (Nutrition Coordinating Center, University of Minnesota, Minneapolis, MN, USA). A total of 150 food and nutrient factors were used in this study (see Supplementary Table 1).

#### DNA methylation analysis

Genomic DNA was extracted from whole blood samples for 374 participants collected at baseline and at 6-year follow-up. DNA extraction was conducted using QIAGEN’s QIAamp DNA Blood Mini Kit (Cat#: 5116); quality control compliance was verified, and the samples were subsequently processed according to the protocol provided by the vendor. For each sample, 250 ng of DNA was used in preparation for Illumina EPIC v2 DNA methylation array analysis by Eurofins Clinical Enterprise Inc. (Framingham, MA, USA). DNA was bisulfite-converted using the EZ DNA Methylation Kit (Zymo Research) following vendor’s instructions. Bisulfite-converted DNA samples were randomly allocated to wells on the Infinium Human MethylationEPIC BeadChip v2, while DNA samples of each participant from baseline and 6-year follow-up were kept on the same array to alleviate batch effects between baseline and 6-year follow up. The following steps included amplification, hybridization, staining, washing, and imaging using the Illumina iScan SQ instrument to obtain raw image intensities. Those intensities were saved as IDAT files for further processing.

Raw IDAT files were processed for QC and normalization using the SeSAMe (version 1.24.0) pipelines [33], a highly recommended method based on systematic evaluation [34]. Briefly, the first normalization was performed using noob background correction [35], then non-linear dye bias corrections was applied. Furthermore, a new *P*-value detection (*P* ≤ 0.05) method by calculating pOOBAH (*P* value with out-of-band array hybridization) was implemented for better correction of background noise [33]. This method accounts for false positives associated with deletions and SNPs confounded by methylation signals. Batch effects were adjusted with ComBat [36] using plate and row as the batch effect. For each methylation CpG site, a beta value was calculated as the ratio of methylated signal intensity to sum of unmethylated and methylated signal intensities. There were 830,880 CpG sites with > 95% call rate used for further analysis.

### Data analysis

#### Specific covariate selection

Covariates for each analysis were chosen based on prior knowledge, published literature, and relevant biological or experimental understanding essential for valid causal inference to minimize spurious associations and overfitting. Our approach focused on identifying exposures associated with diabetes risk while maintaining control over the balance between type I and type II errors. The essential step is to inspect the Q-Q plot to assess whether the models become overfitted when covariates are included.

#### Epigenome-wide association study of incidence of type 2 diabetes and related traits

To identify DNA methylation sites (DMSs) associated with T2D and related outcomes, an epigenome-wide association of T2D and related phenotypes (Hb1Ac, HOMA-IR, HOMA-B) with methylation changes (difference between 6-year follow up and baseline) was performed. Logistic or linear regression models were used with T2D or related traits as outcomes and DNA methylation changes as predictors, adjusting for sex, age, BMI, and cell-type heterogeneity. The composition of six cell-types (neutrophils, monocytes, natural killer cells, B-cells, CD4(+) T-cells, CD8(+) T-cells) in the lymphocytes (white blood cells) were estimated using minfi [37] implemented with R package FlowSorted.Blood.EPIC [38].

Cell-type proportion estimation based on DNA methylation works by comparing the methylation pattern of a whole-blood sample with known reference methylation signatures of purified immune cell types [39]. Each type of immune cell (neutrophils, monocytes, NK cells, B cells, CD4 T cells, and CD8 T cells) exhibits a characteristic methylation pattern at specific CpG sites. Using DNA methylation deconvolution [39], the proportion of each major immune cell type is calculated as a percentage of total leukocytes. The proportions across all estimated cell types sum to 100%. The proportion of each cell type (e.g., percentage of neutrophils, percentage of CD4 T lymphocytes, etc.) is included as a continuous covariate in the regression model. This controls for variation in whole-blood methylation caused by differences in immune cell composition rather than the exposure or phenotype being studied.

The T2D-related phenotypes, Hb1Ac, HOMA-IR, HOMA-B were natural log-transformed to a near-normal distribution [31]. To correct for multiple testing, we set a threshold that satisfied either one of two criteria: epigenome-wide significance *P*-value ≤ 9 × 10^− 8^ as previously described [40] or a false discovery rate (FDR) ≤ 0.05 [41, 42]. To account for X-chromosome inactivation in both women and men, the EWAS was run in women only and in both sexes combined, controlling for confounding variables. However, comparing the QQ plots between the two models suggested that the EWAS with both sexes combined was the most suitable model in terms of minimizing genomic inflation.

#### Association of identified methylation and dietary intake

To identify environmental factors associated with HOMA-IR-associated DMSs, we examined the correlation between identified DMSs with all dietary intakes and lifestyle factors, measured at the 6-year follow-up. All dietary intakes and identified DNA methylation changes were standardized with mean = 0 and standard deviation = 1 for following analyses. For each DMS, DNA methylation changes were modeled as the dependent variable in a linear regression model with each dietary intake and lifestyle factor as a predictor, controlling for sex, age, BMI, smoking status, and supplements at 6-year follow up, and blood cell-type heterogeneity. These association tests were conducted and implemented in SVS (v8.9.0, Bozeman, MT).

For each DMS as a dependent variable, correction for multiple testing was estimated by using the total number of independent variables represented by all dietary intake and lifestyle factors derived by a correlation matrix method [43]. The number of estimated independent factors was 42, representing 150 dietary factors (Supplementary Table 1). Using Bonferroni adjustment for each DMS, we corrected for multiplicity of testing with a *P*-value of 0.05/42, *P* = 0.001.

#### Mediation analysis

To determine if the dietary intake associated with DMSs contributed to insulin resistance through that DMS, a mediation analysis was conducted using the CAUSALMED procedure in SAS 9.4 (SAS, Cary, NC) [44, 45]. Dietary intake and DMS changes were standardized before analysis. Dietary intakes were treated as exposure variables, with HOMA-IR as the outcome, and DMS changes as the mediator. In addition, as age, smoking, BMI, and supplements were associated with DNA methylation, the analyses were adjusted for these variables.

Total, direct, and indirect effects were estimated using mediation analyses. The natural indirect effect (NIE) quantified the effect of dietary factors on HOMA-IR mediated by DMSs, whereas the natural direct effect (NDE) quantified the residual effect not mediated by DMS. The total effect is the overall effect of the exposure on the HOMA-IR without considering the mediator (DMS) [25, 27].

#### Correlation between vitamin B12 and insulin resistance

To examine the correlation between vitamin B12 intake and insulin resistance, the natural logarithm of HOMA-IR was modeled as the dependent variable, while vitamin B12 intake as the predictor was standardized with mean = 0 and standard deviation = 1 using a linear regression model. The association was assessed separately at baseline and at 6-year follow-up; covariates included sex, age, BMI, and smoking status at baseline or at 6-year follow-up. To distinguish between dietary and supplemental vitamin B12, estimated vitamin B12 values were reciprocally adjusted in the model, in addition to the covariates. Untransformed values were used to represent the relationship between HOMA-IR and vitamin B12.

To examine whether serum vitamin B12 levels are associated with insulin resistance, the natural logarithm of HOMA-IR was modeled as the dependent variable, while serum vitamin B12 level as the predictor was standardized with mean = 0 and standard deviation = 1, using a linear regression model adjusted for sex, age, smoking status, and BMI.

#### Mapping DNA methylation signals to gene enhancer regions

DMSs with statistically significant associations to HOMA-IR and dietary vitamin B12 and sugar intake were mapped to human genome build 38. Those coordinates were used to identify enhancer and promoter regions that both contained the CpG methylation site and were predicted to control expression of adjacent and distant genes, using data from GeneHancer [46] and Enhancer Atlas [47]. The transcription factors predicted to bind to the enhancer/promoter were assessed for evidence in published reports of text relevant to the analyzed phenotypes. This action was also performed for the genes near the enhancer/promoter regions whose transcription was predicted to be regulated by those transcription factors. We also queried piRNAdb, a database of PIWI-interacting RNAs and their attributes [https://www.pirnadb.org/, accessed 14 Apr 2025].

## Results

### Participant characteristics

Characteristics of the 374 participants free of T2D at baseline, and with DNA available at both baseline and 6-year follow-up, are shown in Table 1 by 6-year T2D status. Three-quarters of the participants (74.9%) were women, with a mean age of 55.8 years at baseline and 62 years at follow-up. The average follow-up duration from baseline was 6.2 years.

A total of 65 of the 374 participants developed T2D during the follow-up (Table 1). At baseline, participants who did develop T2D were slightly older, with higher BMI, and higher fasting glucose, insulin, HbA1c, and HOMA-IR (*P* < 0.05) when compared to those who did not develop T2D (Table 1). For most of these same characteristics at the 6-year follow-up, the comparisons were similar between T2D and non-T2D participants, with many of those comparisons showing more statistically significant differences. Comparisons between baseline and follow-up (combined) show that insulin, and HOMA-IR tended to decrease (*P* < 0.05), whereas mean fasting glucose, HbA1c, and HOMA-B exhibited insignificant change (*P* > 0.05) over time.

Mean sugar consumption decreased significantly (*P* < 0.05), while total energy intake increased significantly and carbohydrate intake remained unchanged at the 6-year follow-up, compared to baseline. Strikingly, mean vitamin B12 intake at the 6-year follow-up was six times higher than the baseline level. Notably, at the 6-year follow-up, participants without T2D had a vitamin B12 intake three times higher than those who developed T2D (*P* < 0.05). At baseline, no difference in vitamin B12 intake was observed between two groups. There were no significant differences in serum vitamin B12 concentration between participants with T2D or without T2D (future-T2D) at baseline or at 6-year follow-up. Fewer participants were smokers or using alcohol at follow-up than at baseline, but physical activity was unchanged.

In addition, no significant differences were observed between the 374 participants and all participants without T2D (*n* = 873) at baseline for the 17 measures analyzed in this study (*P*-values range from 0.2 to 0.9), suggesting that the 374 participants are highly representative of all participants without T2D at baseline.

### Epigenetic changes and type 2 diabetes and related risk

To identify epigenetic changes that contribute to T2D risk, we performed epigenome-wide association analyses (EWAS) of T2D and related phenotypes at follow-up, including HbA1c, HOMA-IR, and HOMA-B. Genome-wide association analysis with epigenetic changes was performed in relation to incidence of T2D, adjusting for sex, age, BMI, and cell-type heterogeneity. At the epigenome-wide level, no methylation changes were significantly associated with T2D incidence after correction for multiple testing by applying an epigenome-wide significance *P*-value threshold of ≤ 9E-08 or FDR (FDR ≥ 0.05) Similarly, the EWAS for HbA1c and HOMA-B did not detect methylation sites that changed significantly in association over the 6-year period after FDR correction for multiple testing (data not shown).

**Fig. 1:**
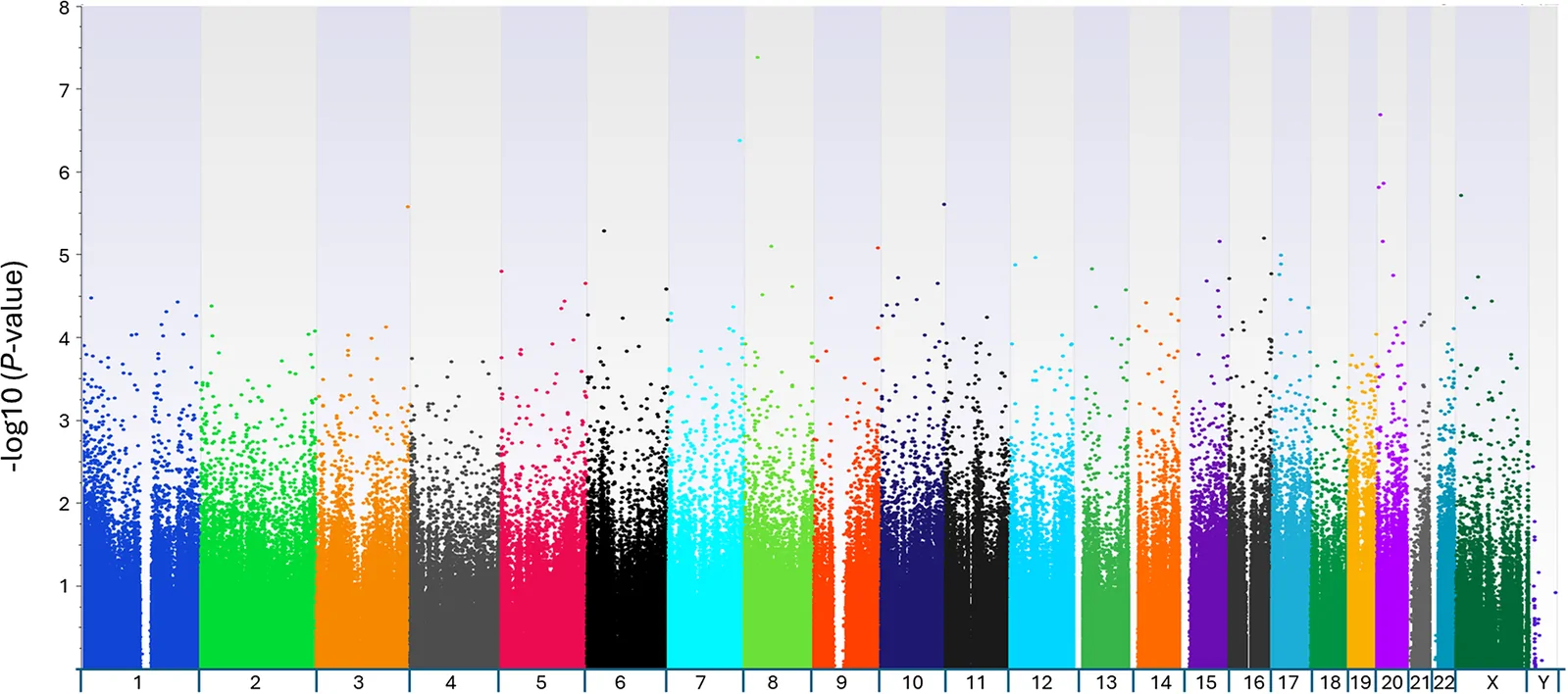
The Manhattan plot of epigenome-wide association study of HOMA-IR. Each point represents a CpG site, plotted by chromosomal position (x-axis) and -log10 (p-value) (y-axis)

**Fig. 2:**
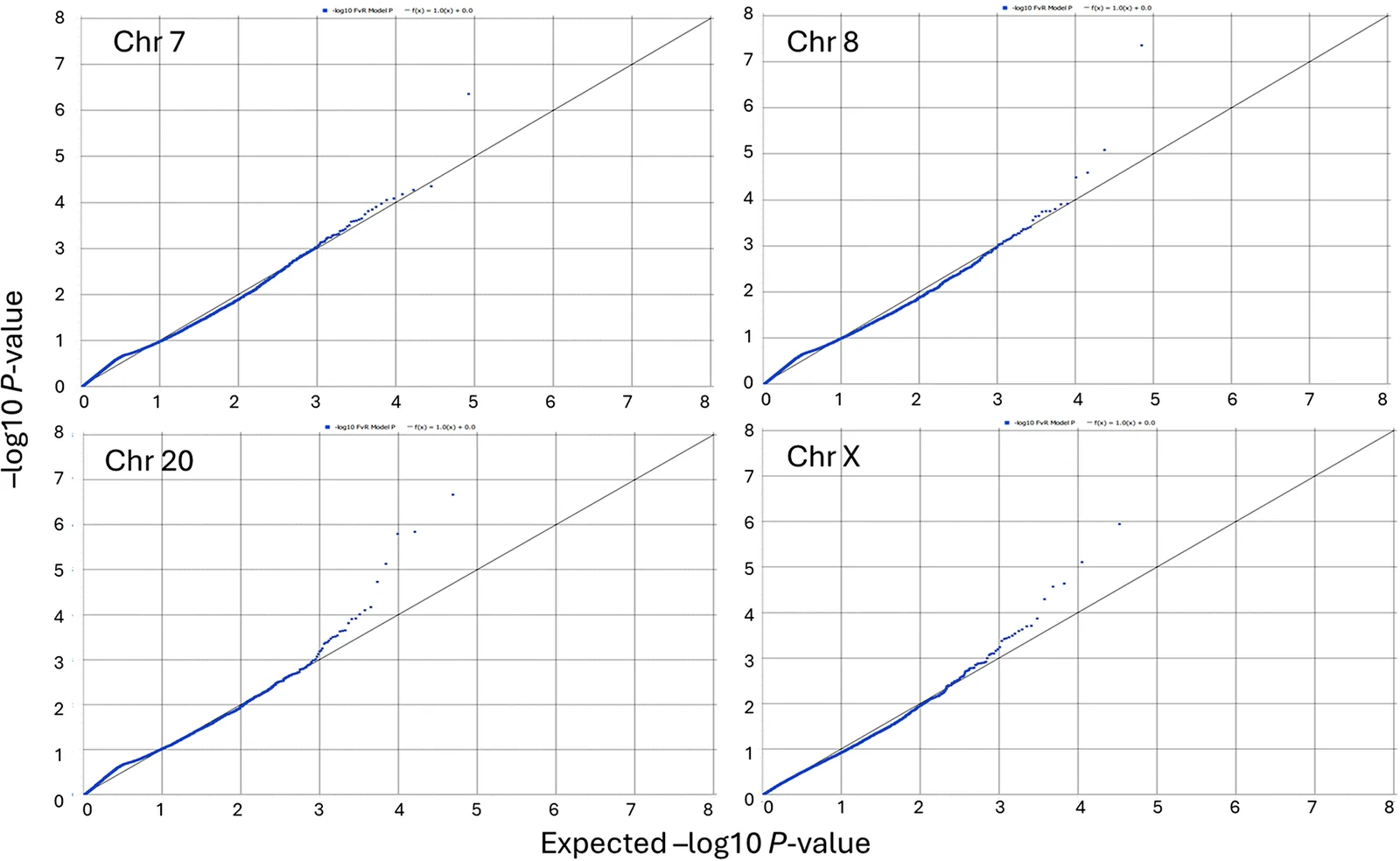
Quantile-quantile (QQ) plot of epigenome-wide association study of HOMA-IR for four chromosomes where DMSs were identified

**Fig. 3:**
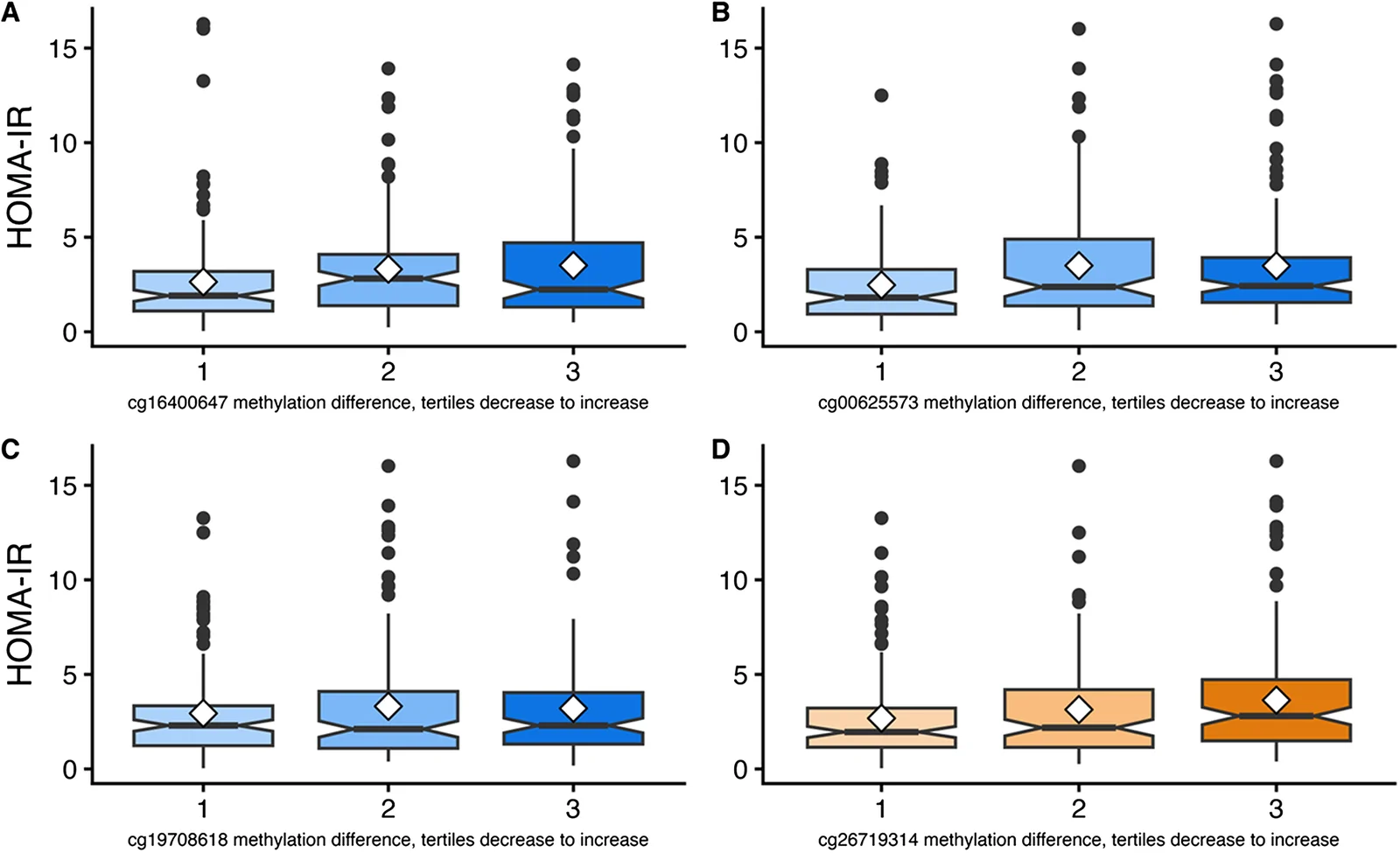
Relationship between DNA methylation at select sites and insulin resistance as mediated by vitamin B12 and total sugar intake. For each panel, delta-DNA methylation values at the indicated site are grouped into tertiles from maximum decrease over the follow-up period to maximum increase. The box plots illustrate median and interquartile ranges plus relevant outliers. The tertile mean is marked by a white diamond. For data plotted in each panel, the respective P values of the natural indirect effect (NIE) are **A**) cg16400647, 0.009; **B**) cg00625573, 0.006; **C**) cg19708618, 0.152; **D**) cg26719314, 0.005. Panels **A**) through **C**) describe the NIE with total dietary vitamin B12, and panel **D**) depicts the NIE with total sugar intake

Seven DMSs were associated with HOMA-IR when a significance threshold at FDR ≤ 0.05 was applied (Fig. 1; Table 2). These significant associations were supported by QQ plots (Fig. 2) and boxplots (Fig. 3). DMS cg16400647 at *REPIN1* (replication initiator 1) was directly associated with insulin resistance (*P* = 4.25E-07, beta = 35.41 ± 6.87). REPIN1 is a pivotal regulator of glucose and insulin sensitivity [48]. DMS cg26665274 at *BMP2* (bone morphogenetic protein 2) was also directly correlated with insulin resistance (*P* = 2.09-07, beta = 12.14 ± 2.29). *BMP2* exhibits specific regulatory functions in adipogenesis [49]. DMS cg19708618 at *LINC01723* was also directly correlated with insulin resistance (*P* = 7.15E-06, beta = 8.98 ± 1.95). In addition, after accounting for X-chromosome inactivation and minimizing genome inflation, cg26719314 on the X chromosome was strongly associated with HOMA-IR. Three other DMSs – cg12344389 near no described gene, cg00625573 near *DNAAF9*, and cg04089779 at *SEL1L2* – were also directly associated with insulin resistance (Table 2).

### Correlation of insulin resistance-associated epigenetic changes and dietary intake

To identify dietary factors associated with HOMA-IR-associated DMSs, we examined correlations between identified DMSs with dietary intake and lifestyle factors, measured at the 6-year follow-up (Table 3). After correction for multiple testing, among 150 dietary factors (Supplementary Table 1) representing 42 independent factors, three DMSs, cg16400647 in *REPIN1* (*P* = 3.10E-05), cg00625573 (*P* = 5.07E-05), and cg19708618 (*P* = 2.61E-05), displayed strong associations with total (dietary plus supplemental) vitamin B12 intake, but not with dietary vitamin B12 intake alone (*P* = 0.93, 0.54, 0.43). The significant correlation between vitamin B12 intake and three DMSs remains even when participants with T2D were excluded from analysis. Importantly, 80% of the vitamin B12 intake at follow-up was from vitamin B12 supplementation, suggesting that B12 from supplements, rather than from food, was the main determinant of the correlation with differential methylation at these three selected sites.

Additionally, changes at cg26665274 of *BMP2* was associated with MUFA 14:1 intake (*P* = 6.15E-04) and saturated fatty 17:0 intake (*P* = 6.15E-04). Furthermore, changes in three other DMSs, cg12344389, cg04876028, and cg26719314 were correlated respectively with alcohol (*P* = 0.019), total alpha-tocopherol (*P* = 4.75E03), and total sugar intake normalized to total energy intake (*P* = 6.37E-03, Table 3). However, these correlations did not reach significance after correction for multiple testing (*P* < 0.001). Furthermore, none of the lifestyle-related factors (physical activities, smoking, alcohol consumption) showed a significant association with these HOMA-IR-associated DMSs.

### Mediation analysis

Given the strong association between the changes in identified DMSs, insulin resistance, and dietary intake, we performed a mediation analysis to examine the potential contributory effects of dietary intake on insulin resistance through DMSs, with dietary intake as exposure, DMSs as mediators, and insulin resistance (HOMA-IR) as the outcome. These analyses were controlled for sex, age, BMI, physical activity, smoking status, and cell type heterogeneity.

Two of three DMSs associated with total vitamin B12 intake, cg16400647 and cg00625573, showed significant natural indirect (mediated) effect (beta = -0.047 and − 0.050, *P* = 0.004 and 0.004, respectively) on decreased insulin resistance (Table 4). Through these two DMSs, vitamin B12 also showed significant total effects (*P* = 0.015 and 0.003) on HOMA-IR. On the other hand, the mediated effect of cg19708618 on insulin resistance induced by vitamin B12 intake did not reach significant (*P* = 0.063), but vitamin B12 intake did exhibit a significant total effect (via cg19708618) on insulin resistance (*P* = 0.005, Table 4).

DMS cg26719314 was shown to mediate direct relationships (*P* = 0.018, beta = 0.037 ± 0.016) between total sugar intake (normalized to total energy) and insulin resistance (HOMA-IR) (Table 4). Despite this, the direct effects of total sugar intake on insulin resistance were not significant (*P* > 0.05).

In the case of three other DMSs (cg12344389, cg26665274, cg04089779) that were correlated with alcohol, MUFA, saturated fat, tocopherol intakes, no significant mediated effect, nor total effects on HOMA-IR were observed for these dietary factors (*P* > 0.05, Supplementary Table 2).

### Correlation between vitamin B12 and insulin resistance

**Fig. 4:**
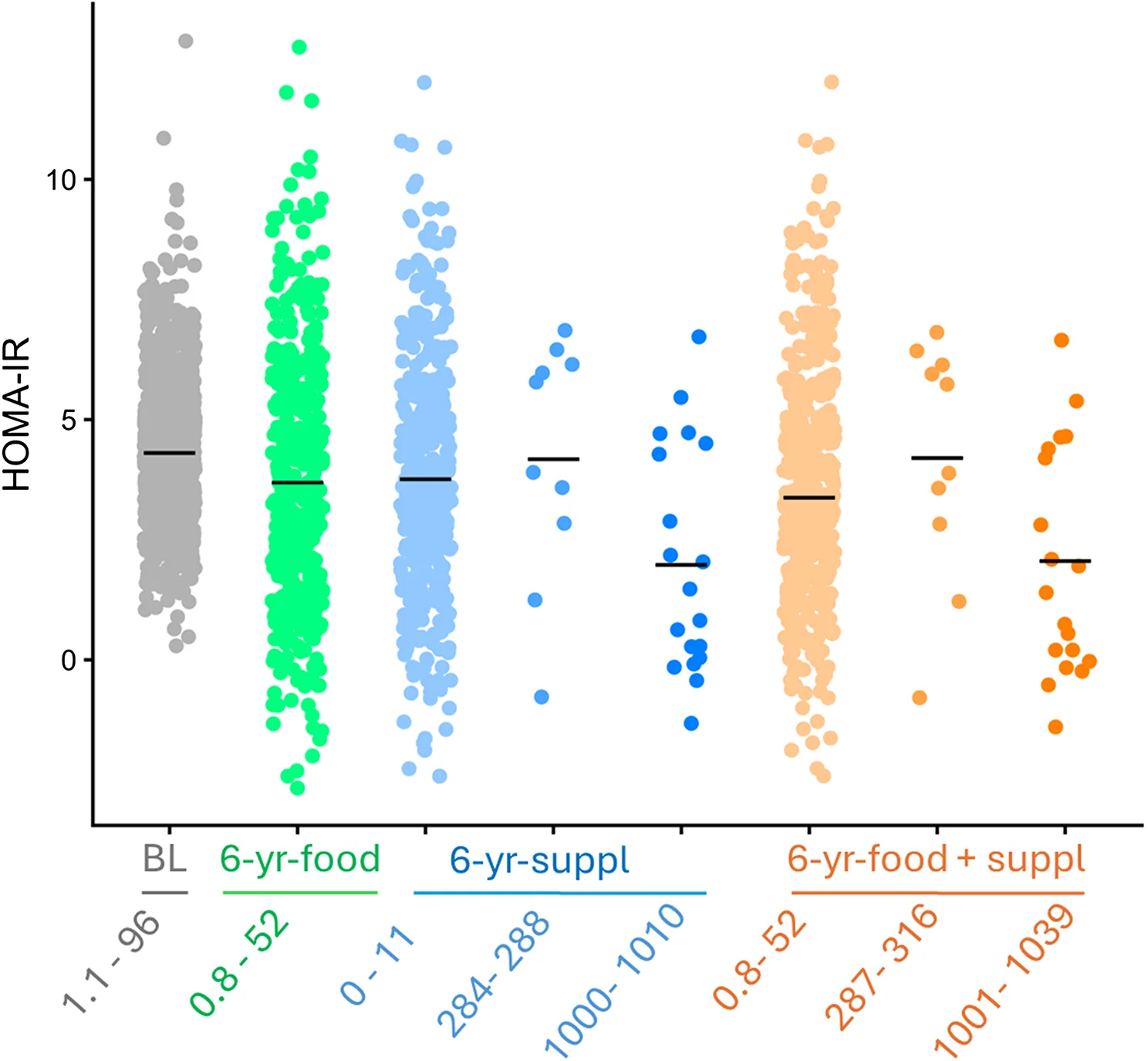
Correlation between dietary and supplemental vitamin B12 intake and HOMA-IR. Vitamin B12 intake was plotted against the estimated HOMA-IR (*y*-axis) for participants who were not using antidiabetic medication at baseline (BL), at 6 years of follow-up considering vitamin B12 only from food (6-yr-food), at 6 years of follow-up considering vitamin B12 only from supplements (6-yr-suppl) in three categories: 0–11, 284–288, 1000–1010 (mcg/day), and at 6 years of follow-up considering vitamin B12 from both food and supplements (6-yr-food+suppl) in three categories: 0.8–52, 287–316, 1001–1039 (mcg/day). Note: vitamin B12 intake categories were defined based on the estimated intake of the vitamin. The three categories reflect three sources of vitamin B12: food, multivitamin supplements, and high-dose vitamin B12 supplements. The black horizontal line segments indicate the mean HOMA-IR value for each group. All HOMA-IR values were calculated from linear regression models after adjustment for sex, age, BMI, smoking status. For 6-yr-food, HOMA-IR values were adjusted additionally for vitamin B12 intake from supplements

As in mediation analysis demonstrated that vitamin B12 intake contributed to decreased insulin resistance through epigenetic changes, we next examined whether vitamin B12 intake correlated with insulin resistance across the entire cohort. As anti-diabetes medication affects insulin resistance, the correlation was examined only in participants who were not taking anti-diabetic medication. At baseline, total vitamin B12 intake (from food and supplements) was not significantly associated with insulin resistance (*P* = 0.6, estimate=-0.012 ± 0.023, *n* = 953) (Table 5; Fig. 4). At the 6-year follow-up (Fig. 4), vitamin B12 either from supplementation alone or total vitamin B12 intake (from food and supplements) was significantly correlated with decreased insulin resistance (*P* = 0.011 and estimate= -0.133 ± 0.044, *P* = 0.01 and estimate=-0.114 ± 0.044, respectively). But dietary vitamin B12 intake (from food at 6-year follow-up) only did not reach significance (*P* = 0.103, estimate = -0.07 ± 0.043) while adjusted for supplementary vitamin B12 intake. When participants who used anti-diabetes medication were included in the analysis, the correlation between vitamin B12 intake and HOMA-IR remained the same (data not shown). Furthermore, there is no association between baseline vitamin B12 intake and insulin resistance in participants who did not use anti-diabetes medication at the 6-year follow-up (*P* = 0.283, *n* = 415).

To assess whether serum vitamin B12 level is associated with insulin resistance, we first examined the correlation between serum B12 levels and dietary B12 intake among participants who did not use anti-diabetes medication. After adjusting for sex, age, BMI, and smoking status, serum B12 showed a strong correlation with dietary B12 intake at both baseline and the 6-year follow-up (*P* < 0.0001 and < 0.0001; beta = 3.86 ± 1.0 and 0.39 ± 0.07, respectively). In contrast, no association was observed between serum B12 and insulin resistance in participants who did not use anti-diabetes medication at either time point (*P* = 0.997 and 0.959, respectively).

### Mapping DNA methylation signals to gene enhancer regions

Because DNA methylation sites regulate gene expression, we determined which identified CpGs map within enhancer elements that regulate activity of nearby genes. Two large catalogs of enhancer/promoter data served this purpose: GeneHancer [46] and Enhancer Atlas [47]. CpG cg26665274 on chromosome 20 maps to GeneHancer enhancer element GH20J006764. This enhancer is predicted to regulate six genes: *BMP2*, *LOC105372517*, *ENSG00000302641*, *HSALNG0128484*, *LINC01713* and *FERMT1* within a 705-kb region (Supplementary Fig. 2, top section). Numerous transcription factors (TFs) and regulators are predicted to bind to this enhancer, including TCF7L2, HNF4A, CTCF, YY1, PKNOX1, ZFP36 and EZH2 (Supplementary Table 2). Many of these TFs have literature evidence connecting them to insulin and its functions. Chromosome X CpG cg26719314 maps to GeneHancer enhancer element GH0XJ013211. This enhancer is predicted to control five genes: *lnc-ATXN3L-5*, *piR-1510* (*piR-31534*), *ENSG00000309443*, *ATXN3L* and *FAM9C* in a region of 306 kb (Supplementary Fig. 2, bottom section). The enhancer element likely binds TFs EP300, EZH2 and JUND.

Vitamin B12 enhancer regions: CpG cg16400647 on chromosome 7 maps to GeneHancer enhancer element GH07J150372. This enhancer regulates 18 genes, including *REPIN1*, *NUB1*, *CHPF2*, *TMEM176A*, several zinc finger family members, and lncRNAs. At least one fifth of TFs assigned to this enhancer, including TCF7L2, PPARA, HNF1A, HNF4A, REPIN1, FOXA2 and BCL11A, have been implicated in T2D risk, insulin action, or are targets of T2D medication. CpG cg19708618, which maps to chromosome 20, resides within GeneHancer enhancer element GH20J012950. This enhancer affects four genes: *LINC01723*, *LINC01722*, *ENSG00000269931* and *SPTLC3*. Specific TFs have not been assigned to this 60-bp segment. CpGs cg12344389, cg00625573 and cg04089779 do not map within cataloged enhancer regions at GeneHancer. No CpG mapped within cataloged enhancer regions at GeneHancer, but have evidence from the Enhancer Atlas for enhancers that are active in two, 64 and 30 cell lines, respectively. CpGs cg00625573 and cg04089779 reside in enhancers that are active the same 22 cell lines.

## Discussion

This 6-year longitudinal study of older Puerto Rican adults living in Massachusetts, investigated epigenetic changes in 374 participants free of T2D at baseline, of which 65 developed the disease during the follow-up period. While our analyses did not reveal epigenome-wide significant changes directly linked to T2D or HbA1c, they did identify seven DMSs significantly associated with insulin resistance after correcting for multiple testing. Further analyses revealed that three DMSs were strongly correlated with vitamin B12 intake sourced primarily from supplements. X chromosome DMS cg26719314 showed a significant association with total sugar intake. Mediation analysis suggested that vitamin B12 intake might help decrease insulin resistance through its influence on three specific methylation sites including *REPIN1*. Conversely, high total sugar intake appears to increase insulin resistance, mediated by the epigenetic changes at cg26719314.

To learn how environmental factors contribute to T2D risk, we used a unique, three-step approach in our longitudinal cohort study to pinpoint epigenetic changes in the period of six years. First, an EWAS identified epigenetic changes associated with insulin resistance within the longitudinal cohort. Next, we determined which environmental factors correlated with the DMSs identified in the EWAS. Finally, we used mediation analysis to assess if these associated environmental factors contributed to the study outcome (insulin resistance) through the epigenetic markers. We successfully applied this procedure to identify three DMSs linked to vitamin B12 intake, and one to total sugar intake. These epigenetic loci appear to mediate the contribution of these dietary factors to insulin resistance. While we identified such connections with B12 and sugar, we did not establish associated environmental factors for the other three epigenetic loci. A plausible reason for this is numerous other environmental factors were not measured, such as all aspects of diet, lifestyle, living environment (e.g., population density and population exposures), or sleep patterns.

### Total sugar intake and insulin resistance

It is well established that total sugar and refined carbohydrate intakes are dietary risk factors for T2D [50]. In the present study, we identified a specific DMS, cg26719314 on the X chromosome, that was associated with both insulin resistance and total sugar intake. Mediation analysis further showed that increased total sugar intake contributes to increased insulin resistance, with cg26719314 mediating this effect. Interestingly, although there is a mediated effect, the overall “total effects” of total sugar intake on insulin resistance were not statistically significant. This suggests that sugar intake may contribute to insulin resistance mainly through mediation of this epigenetic change. This proposes new areas of investigation to define complex interplay between diet, epigenetics, and insulin resistance.

### Vitamin B12 and insulin resistance

Studies have linked vitamin B12 deficiency to increased insulin resistance [51], and metformin use is known to reduce plasma vitamin B12 concentration [52]. Similarly, higher intake of vitamin B12 has been correlated with reduced insulin resistance [53]. However, the exact molecular mechanisms behind these associations have not been described fully. We pinpointed three differentially methylated sites (DMSs) where epigenetic changes were associated with both insulin resistance and vitamin B12 intake. Further investigation with mediation analysis, revealed that vitamin B12 supplementation, and not B12 from food, contributed to a reduction in insulin resistance through two of these three DMSs.

An obvious concern is whether vitamin B12 supplementation is the result of reverse causality in T2D. To address this, we examined the correlation between vitamin B12 supplementation (as a binary variable) and T2D incidence. The results showed that vitamin B12 supplementation was independent of T2D status (Chi-square, *P* = 0.554), eliminating the possibility of reverse causation. On the other hand, several observational studies suggested higher serum B12 often appears alongside higher glucose levels [54] or greater glucose fluctuations [55]. We showed serum vitamin B12 was not associated with insulin resistance either at baseline or at 6-year follow-up in this study. The high serum B12 in previous reports could imply elevated B12 is likely a consequence of dysregulated glucose metabolism or related metabolic conditions, not necessarily a driver (a cause of T2D).

Folate and homocysteine appear as key intermediaries between vitamin B12 status and insulin resistance. Vitamin B12 deficiency has been linked to both insulin resistance and elevated homocysteine [56, 57]. Additionally, in people with extreme obesity, both folate and vitamin B12 status were associated with insulin resistance, notably as low B12/high folate [58]. Specifically, individuals with the highest HOMA-IR values were also in the group with the lowest serum B12 levels (< 200 pmol/L) and highest plasma folate (> 10.2 nmol/L). B12 deficiency potentially drives sub-optimal folate metabolism and elevates folate concentration in plasma and red blood cells. Unusually high levels of methylmalonic acid (MMA) caused by disruptions in its conversion to succinyl-CoA, a reaction driven in part by methylmalonyl-CoA mutase and B12 as its co-enzyme, also have been linked to greater insulin resistance and lipogenesis [58]. Moreover, imbalances in vitamin B12, homocysteine and folate could affect the transsulfuration pathway, with effects on glutathione synthesis and cysteine oxidation [59]. This pathway has been implicated as a contributor to insulin resistance, hepatic steatosis, circulatory impairment, oxidative stress, and inflammation [59].

### REPIN1 and insulin resistance

From the mediation analysis, vitamin B12 appears to contribute to reduced insulin resistance mediated through decreased cg16499647 methylation at *REPIN1.* This gene encodes a zinc-finger transcription factor [60]. Its expression in adipose tissue is markedly reduced in hyperinsulinemic rats [57]. In mice, Repin1 deficiency improved glucose metabolism and reduced adipose tissue mass and inflammation [61], while an adipose-tissue knockout of *Repin1* improved whole-body insulin resistance and lipid metabolism [62]. Human studies showed that a 12-bp deletion at *REPIN1* was associated with reduced insulin resistance (HOMA-IR) and low risk of T2D [48]. Interestingly, the 12-bp deletion induced higher expression of *REPIN1*, and high protein expression of facilitated glucose transporter SLC2A2 and thrombospondin/fatty acid receptor CD36 in in-vitro studies [48]. This observation supports our finding that vitamin B12 contributes to reduced insulin resistance mediated through decreased cg16499647 methylation at *REPIN1*. Hence, the most likely interpretation of these results is the *REPIN1* locus is the target of vitamin B12 resulting in differential methylation at CpG cg16400647. Beyond this mediated effect of three identified DMSs, vitamin B12 also demonstrated a significant total effect on insulin resistance, suggesting it influences insulin sensitivity through other pathways as well. Notably, the mediated effects we observed were specifically linked to the *REPIN1* gene.

### Potential influences of other identified DMSs on T2D

In addition to the DMSs described above, another identified DMS, cg26665274 at bone morphogenic protein 2 (*BMP2*), is highly relevant to insulin resistance and T2D risk. In hypertrophic obesity, BMP2 and BMP4 are signaling molecules that regulate adipocyte development and differentiation. Specifically, an increase in *BMP2* expression is observed in visceral white adipose tissue, but not in subcutaneous adipose tissue, and in overweight or obese individuals, but not in lean individuals [63]. BMP2 and BMP4 inhibit insulin signaling and affect beta cell function, leading to insulin resistance [64, 65]. In patients with diabetes, BMP2 hinders beta cell function and proliferation [66]. Select members of the BMP family have been described as insulin sensitizers [67]. BMP2 and BMP6 were shown to enhance insulin-mediated glucose uptake in adipocytes that were either insulin-sensitive or -insensitive via induction of PPARG [67]. Evidence shows that the effects of BMP2 and BMP6 overlap but are not completely redundant. These effects include regulating genes that function in glucose and fatty acid metabolism as well as the adipokines leptin (LEP) and visfatin (NAMPT) [67]. Notably, the affected genes include facilitated glucose transporters SLC2A1 and SLC2A4.

DMS cg04089779 maps near the *SEL1L2* gene. The SEL1L2 protein is an adaptor subunit of SYVN1 ubiquitin ligase and has been demonstrated to bind to the ubiquitinated form of STXBP5L, along with Hrd-1 (SYVN1), OS9 and SEL1L. STXBP5L, also known as tomosyn-2, is a syntaxin binding protein that shows progressive ubiquitination with increasing levels of glucose in cell culture which, in turn, enhances turnover of STXBP5L. Furthermore, phosphorylation and ubiquitin-driven degradation of STXBP5L suppress insulin secretion [68]. In the Interval and LonGenity cohorts, both REPIN1 and SEL1L2 showed significant associations with aging [69].

DMS cg00625573, associated with vitamin B12 intake, is located near *DNAAF9*, encoding a dynein axonemal assembly factor. *DNAAF9* is one of 107 proteins downregulated in liver cells after atorvastatin treatment [70]. This is noteworthy given that atorvastatin has been documented to be diabetogenic [71], an effect not unique to this statin [72] and one that likely is mediated, in part, via DNA methylation [73]. Taken together, the diet-sensitive DNA methylation sites identified in this study have important implications for building mathematical models that are designed to predict risk of incident T2D.

### Limitations

Our study offers valuable insights but does have certain limitations. First, we relied on blood DNA to assess epigenetic changes. While convenient, blood may not always be the ideal tissue to capture the most relevant epigenetic signals related to diet, lifestyle, and insulin resistance. Other tissues involved in metabolism might offer more direct interrogation. Second, although this was a longitudinal study, its average duration was 6.2 y. Longer follow-up in other studies could better capture the dynamic changes in environmental factors and their long-term epigenetic impacts. Third, while we examined several dietary and lifestyle factors, the development of insulin resistance is influenced by a multitude of environmental factors. Our study collected and analyzed only a limited number of these factors. Future research could benefit from a more comprehensive assessment of environmental exposures. Finally, the modest sample size of our study, particularly the small number of participants who developed T2D, suggests that our findings should be interpreted with caution. A larger sample size with increased statistical power to detect DMSs and environmental factors would strengthen our results. Further studies could include randomized, double-blind supplementation trials which could help confirm whether B12 alters specific methylation sites and lower insulin resistance. In vitro experiments using cells from various tissues could assess how vitamin B12–related DNA methylation changes influence gene expression and transcription factor binding. Together, these approaches would help determine whether higher B12 status consistently predicts lower insulin resistance and identify subgroups most likely to benefit from targeted interventions.

## Conclusion

Our findings indicate that vitamin B12 supplementation and sugar intake influence the risk of developing T2D through specific epigenetic changes at loci with mechanistic links to insulin resistance. This study demonstrated epigenetic connections between dietary habits and the development of T2D, and has important implications for the development of precision nutrition strategies for the prevention of type 2 diabetes.

**Table 1:** General characteristics of participants at baseline and 6-year follow-up

		Baseline			6-Year follow-up		
		Future-T2D	Non-T2D	Combined	T2D	Non-T2D	Combined
N		65	309	374	65	309	374
Female (%)		49 (75.4%)	231 (74.8%)	280 (74.9%)			
Age (SD)		57.9 (7.8)*	55.4 (7.2)	55.8 (7.3)	64.3 (7.9)*	61.6 (7.4)	62.0 (7.5)
Body mass index (BMI, SD)		32.6 (6.6)*	30.0 (6.2)	31.0 (6.3)	31.7 (6.0)*	29.8 (6.1)	30.2 (6.1)
Type 2 diabetes		0	0	0	65	0	65
Fasting glucose (mg/dl)		102.4 (12.2)*	95.9 (9.8)	97.1 (10.5)	118.8 (29.4)*	92.7 (11.6)	97.3 (18.9)
Insulin (µIU/ml)		17.7 (12.0)*	14.0 (9.3)	14.7 (9.9)	17.5 (13.1)*	11.2 (9.3)	12.3 (10.4)**
Glycosylated hemoglobin A1c (HbA1c, %)		6.5 (0.7)*	6.0 (0.7)	6.1 (0.7)	6.8 (1.1)*	5.8 (0.5)	6.0 (0.7)
serum vitamin B12 (pg/ml)		555.5 (274.2)	532.5 (250.5)	536.4 (254.4)	586.3 (289.4)	565.6 (305.8)	569.2 (302.7)
HOMA-IR		4.6 (3.6)*	3.4 (2.4)	3.6 (2.7)	5.2 (3.8)*	2.7 (2.4)	3.1 (2.9)**
HOMA-B		172.1 (116.7)	157.7 (103.3)	160.2 (105.7)	209.5 (705.8)	149.4 (163.8)	159.9 (10.4)
Smoker (n, %)	Current smoker	15 (23.1%)	79 (25.6%)	94 (25.1%)	9 (13.8%)*	66 (21.4%)	75 (20.1%)
	Past smoker	19 (29.2%)	81 (26.2%)	100 (26.7%)	25 (38.5%)	98 (31.7%)	123 (32.9%)
	Never smoker	31 (47.7%)	148 (47.9%)	179 (47.9%)	31 (47.9%)	142 (46.0%)	173 (46.3%)
Alcohol use (n, %)	Current drinker	20 (30.8%)*	155 (50.2%)	175 (46.8%)	11 (16.9%)*	94 (30.4%)	105 (28.1%)**
	Past drinker	22 (33.8%)*	66 (21.4%)	88 (23.5%)	31 (47.7%)	133 (43.0%)	164 (43.9%)**
	Never drinker	23 (35.4%)	87 (28.2%)	110 (29.4%)	23 (35.4%)*	82 (26.5%)	105 (28.1%)
Physical activity score		31.8 (4.5)	31.9 (4.6)	31.8 (4.6)	31.3 (5.7)	32.0 (6.25)	31.9 (6.1)
Vitamin B12 (mcg/d)		10 (7.3)	11 (8.5)	10.8 (8.3)	22.9 (126)*	74.7 (238)	65.7 (223)**
Total sugar intake (g/d)		123 (93)	117 (68)	118.2 (72.6)	109.9 (82)	116.3 (76.9)	115.2 (77.8)**
Total sugar intake (% of total energy)		21.3 (8.1)	20.7 (7.5)	20.8 (7.6)	17.3 (6.4)	17.2 (5.9)	17.2 (6.0)
Total carbohydrate intake (g/d)		298 (164)	287 (121)	289 (129)	282 (151)	300 (148)	297 (149)
Total energy intake (kcal, SD)		2305 (1242)	2278 (974)	2283 (1024)	2414 (1198)*	2599 (1257)	2567 (1247)**

*P-value < 0.05 significant difference between non-T2D and T2D at baseline or 6-year follow-up

**P < 0.05 significant difference between baseline and 6-year follow-up

**Table 2:** EWAS identified epigenetic changes associated with insulin resistance

DMS	Chr	Position	Gene	*P*-value*	Beta	SE	FDR
cg16400647	7	150,372,618	*REPIN1*	4.25E-07	35.41	6.87	0.018
cg12344389	8	27,421,801	*intergenic region*	4.26E-08	14.61	2.61	0.001
cg00625573	20	3,405,073	*DNAAF9*	1.56E-06	7.73	1.58	0.012
cg26665274	20	6,766,762	*BMP2*	2.09E-07	12.14	2.29	0.005
cg19708618	20	12,950,305	*LINC01723*	7.15E-06	8.87	1.95	0.043
cg04089779	20	13,882,559	*SEL1L2*	1.41E-06	12.84	2.62	0.017
cg26719314	X	13,212,234	*LINC02154*	1.95E-06	11.25	2.32	0.032

*Epigenome association analyses of insulin resistance were performed using linear regression models adjusted for age, sex, BMI and cell-type heterogeneity

Significant threshold for correction for multiple testing is *P* ≤ 9E-08 or FDR ≤ 0.05

HOMA-IR (insulin resistance) was transformed using the natural logarithm

**Table 3:** Correlation between HOMA-IR associated epigenetic changes and dietary factors

DMS^$^	Nutrient^$^	*P*-value^#^	beta	SE
cg16400647	Vitamin B12*	3.10E-05	-0.216	0.051
cg12344389	Alcohol**	1.88E-02	-0.208	0.088
cg00625573	Vitamin B12*	5.07E-05	-0.205	0.050
cg19708618	Vitamin B12*	2.61E-05	-0.221	0.052
cg26665274	MUFA 14:1 (myristoleic acid)**	6.15E-04	0.278	0.081
	SFA 17:0 (margaric acid)**	1.01E-03	0.293	0.088
cg04089779	Synthetic alpha-tocopherol **	4.23E-03	0.143	0.050
	Total alpha –tocopherol equivalents**	4.75E-03	0.142	0.050
cg26719314	Total sugar %**	6.37E-03	0.159	0.058
	MUFA 20:1 (gadoleic acid)**	8.11E-03	-0.286	0.107

*Adjusted for cell-type, age, sex, education, BMI, smoking, total energy intake at 6-year follow-up

**Adjusted for cell-type, age, sex, education, BMI, smoking status, total energy intake, vitamin B12 supplementation at 6-year follow-up

^#^Significant threshold for correction multiple testing is *P* ≤ 0.001

^$^DMS changes and nutrients were standardized

**Table 4:** Direct and indirect effect of vitamin B12 and total sugar intake on insulin resistance with four DMSs as mediators

DMS^$^	Nutrient^$^	Effect	Estimate	Standard Error	95% CI		Z	*P*-value*
cg16400647	Vitamin B12	Total Effect	-0.122	0.050	-0.221	-0.023	-2.420	0.015
	(mcg/day)	Controlled Direct Effect (CDE)	-0.078	0.048	-0.172	0.015	-1.640	0.101
		Natural Direct Effect (NDE)	-0.075	0.049	-0.170	0.020	-1.540	0.123
		Natural Indirect Effect (NIE)	-0.047	0.016	-0.080	-0.015	-2.870	0.004
		Percentage Mediated	38.7	17.9	3.7	73.8	2.170	0.030
cg00625573	Vitamin B12	Total Effect	-0.146	0.050	-0.244	-0.049	-2.940	0.003
	(mcg/day)	Controlled Direct Effect (CDE)	-0.097	0.047	-0.189	-0.005	-2.060	0.039
		Natural Direct Effect (NDE)	-0.096	0.047	-0.189	-0.003	-2.030	0.043
		Natural Indirect Effect (NIE)	-0.050	0.017	-0.085	-0.016	-2.880	0.004
		Percentage Mediated	34.3	13.9	7.1	61.6	2.47	0.014
cg19708618	Vitamin B12	Total Effect	-0.152	0.055	-0.259	-0.045	-2.790	0.005
	(mcg/day)	Controlled Direct Effect (CDE)	-0.103	0.047	-0.194	-0.011	-2.200	0.028
		Natural Direct Effect (NDE)	-0.103	0.047	-0.196	-0.011	-2.180	0.029
		Natural Indirect Effect (NIE)	-0.049	0.027	-0.101	0.003	-1.860	0.063
		Percentage Mediated	32.3	15.3	2.3	62.3	2.11	0.035
cg26719314	Total sugar %	Total Effect	0.081	0.049	-0.014	0.177	1.670	0.095
	(of total energy kcal)	Controlled Direct Effect (CDE)	0.047	0.048	-0.047	0.141	0.980	0.325
		Natural Direct Effect (NDE)	0.044	0.048	-0.050	0.139	0.920	0.358
		Natural Indirect Effect (NIE)	0.037	0.016	0.006	0.067	2.370	0.018
		Percentage Mediated	45.4	30.2	-13.8	104.6	1.500	0.133

*Adjusted for sex, age, smoking status, BMI and total energy intake. Significant threshold is *P* ≤ 0.05

^$^DMS changes and nutrients were standardized

HOMA-IR (insulin resistance) was transformed using the natural logarithm

**Table 5:** Correlation between vitamin B12 intake and HOMA-IR in participants without use of diabetes medication

	Vitamin B12 source	*N*	Estimate	SE	*P*-value*
Baseline	Food and supplement	953	-0.012	0.023	0.600
6-year follow-up	Food only	415	-0.070	0.043	0.103^#^
	Supplement only	415	-0.113	0.044	0.011
	Food and supplement	415	-0.114	0.044	0.010

*Adjusted for sex, age, BMI, smoking status

^#^Adjusted for sex, age, BMI, smoking status, supplementary vitamin B12

Significant threshold is *P* ≤ 0.05

HOMA-IR (insulin resistance) was transformed using the natural logarithm

## Supplementary Information

Below is the link to the electronic supplementary material.

Supplementary Material 1: Supplementary Fig. 1: The flowchart of the study design. Supplementary Fig. 2: Representation of genomic regions containing the HOMA-IR DMS signals on Chr 20 and X and their enhancers. The upper figure represents chromosome 20 and cg26665274, and the lower figure shows chromosome X and cg26710314. For chr 20, DMS cg26665274 (yellow dot) maps within enhancer GH20J006764 (purple box) and just upstream of BMP2, a gene implicated in adipogenesis and insulin signaling (dark blue box). Other genes depicted in light blue are regulated by enhancer GH20J006764. Nearby genes not regulated by the enhancer are illustrated in green. For chr X (lower figure), DMS cg26665274 (yellow dot) maps within enhancer GH0XJ013211 (purple box). Genes proposed to be regulated by this enhancer are drawn in light blue and those not regulated by the enhancer are illustrated in green. Genes drawn above the genome segment (thick black line) are transcribed left to right, while those below this segment are transcribed right to left. Numbers indicate positions in megabases in genome build GRCh38/hg38. Abbreviations: E1, ENSG00000302641; E3, ENSG00000309443; GS1, GS1-600G8.3; H4, HSALNG0128484; L4, LOC105373134; L7, LOC105372517. Supplementary Table 1: 150 daily food and nutrient factors. Supplementary Table 2: Transcription factors predicted to bind to enhancer elements containing selected differential methylation sites.

## Data Availability

The data described in the manuscript will be available under sharing agreement in alignment with Institutional Review Board policies and regulations of University of Massachusetts Lowell and Tufts University and approval by the local research committee.

## Abbreviations

BMP2: Bone morphogenic protein 2
BPRHS: Boston Puerto Rican Health Study
DMSs: Differentially DNA methylated sites
EWAS: Epigenome-wide Association Study
FDR: False discovery rate
FFQ: Food frequency questionnaire
HbA1c: Glycosylated hemoglobin
HOMA-IR: Homeostatic model assessment of insulin resistance
HOMA-B: Homeostatic model assessment of β-cell function
NIE: Natural indirect effect
NDE: Natural direct effect
QC: Quality control
REPIN1: Replication initiator 1
SeSAMe: SEnsible Step-wise Analysis of DNA MEthylation BeadChips
T2D: Type 2 diabetes

## Declarations

### Disclaimer

Mention of trade names or commercial products in this publication is solely for the purpose of providing specific information and does not imply recommendation or endorsement by the US Department of Agriculture and the National Institutes of Health or the other funders. These entities had no part in the design of this project, collection, analysis, and interpretation of data, nor in composing the manuscript.
