# Supplementary material for: Epigenetic changes reveal that vitamin B12 intake is associated with lower insulin resistance in older adults
Source: Genes Nutr. 2026 Aug 4;21:26. doi: 10.1186/s12263-026-00813-z (PMC13721465; doi:10.1186/s12263-026-00813-z)
Supplement: Supplementary file 1 [file 12263_2026_813_MOESM1_ESM.docx]

| **Supplementary Table 1. List of daily nutrient intake** | |  |
| --- | --- | --- |
| Nutrient group | nutrient | unit |
| alcohol | % Calories from Alcohol | % |
| alcohol | Alcohol | g |
| amino acids | Betaine | mg |
| amino acids | 3-Methylhistidine | mg |
| amino acids | Alanine | g |
| amino acids | Arginine | g |
| amino acids | Aspartic Acid | g |
| amino acids | Cystine | g |
| amino acids | Glutamic Acid | g |
| amino acids | Glycine | g |
| amino acids | Histidine | g |
| amino acids | Isoleucine | g |
| amino acids | Leucine | g |
| amino acids | Lysine | g |
| amino acids | Methionine | g |
| amino acids | Phenylalanine | g |
| amino acids | Proline | g |
| amino acids | Serine | g |
| amino acids | Threonine | g |
| amino acids | Tryptophan | g |
| amino acids | Tyrosine | g |
| amino acids | Valine | g |
| ash | Ash | g |
| caffeine | Caffeine | mg |
| carbohydrates | % Calories from Carbohydrate | % |
| carbohydrates | Added Sugars (by Total Sugars) | g |
| carbohydrates | Added Sugars (by Available Carbohydrate) | g |
| carbohydrates | Available Carbohydrate | g |
| carbohydrates | Fructose | g |
| carbohydrates | Galactose | g |
| carbohydrates | Glucose | g |
| carbohydrates | Lactose | g |
| carbohydrates | Pectins | g |
| carbohydrates | Starch | g |
| carbohydrates | Sucrose | g |
| carbohydrates | Tagatose | mg |
| carbohydrates | Total Carbohydrate | g |
| carbohydrates | Total Sugars | g |
| carbohydrates | % calorie from total sugar |  |
| energy | Energy (kilocalories) | kcal |
| energy | Energy (kilojoules) | kj |
| Fat | % Calories from Fat | % |
| fat | Solid Fats | g |
| fat | Total Fat | g |
| fiber | Insoluble Dietary Fiber | g |
| fiber | Soluble Dietary Fiber | g |
| fiber | Total Dietary Fiber | g |
| Food | Total grams | g |
| food | Glycemic Index (bread reference) | ? |
| food | Glycemic Index (glucose reference) | ? |
| food | Glycemic Load (bread reference) | ? |
| food | Glycemic Load (glucose reference) | ? |
| food | Gram amount of food | ? |
| Grain | Refined Grains (ounce equivalents) | oz equiv |
| Grain | Total Grains (ounce equivalents) | oz equiv |
| grain | Whole Grains (ounce equivalents) | oz equiv |
| lignans | Pinoresinol | mcg |
| lignans | Secoisolariciresinol | mcg |
| lignans | Total Lignans | mcg |
| Lipid | Cholesterol | mg |
| Lipid | Cholesterol to Saturated Fatty Acid Index | ? |
| minerals | Acesulfame Potassium | mg |
| minerals | Calcium | mg |
| minerals | Copper | mg |
| minerals | Iron | mg |
| minerals | Magnesium | mg |
| minerals | Manganese | mg |
| minerals | Phosphorus | mg |
| minerals | Potassium | mg |
| minerals | Selenium | mcg |
| minerals | Sodium | mg |
| minerals | Zinc | mg |
| MUFA | % Calories from MUFA | % |
| MUFA | MUFA 14:1 (myristoleic acid) | g |
| MUFA | MUFA 16:1 (palmitoleic acid) | g |
| MUFA | MUFA 18:1 (oleic acid) | g |
| MUFA | MUFA 20:1 (gadoleic acid) | g |
| MUFA | MUFA 22:1 (erucic acid) | g |
| natatoc | Natural Alpha-Tocopherol (RRR-alpha-tocopherol or d-alpha-tocopherol) | mg |
| natural and chemical compounds | Lariciresinol | mcg |
| natural and chemical compounds | Matairesinol | mcg |
| natural and chemical compounds | Biochanin A | mg |
| natural and chemical compounds | Coumestrol | mg |
| natural and chemical compounds | Daidzein | mg |
| natural and chemical compounds | Formononetin | mg |
| natural and chemical compounds | Formononetin | mg |
| natural and chemical compounds | Genistein | mg |
| natural and chemical compounds | Glycitein | mg |
| natural and chemical compounds | Inositol | g |
| natural and chemical compounds | Mannitol | g |
| natural and chemical compounds | Oxalic Acid | mg |
| natural and chemical compounds | Phytic Acid | mg |
| natural and chemical compounds | Pinitol | g |
| natural and chemical compounds | Sucrose Polyester | g |
| nitrogen | Nitrogen | g |
| Protein | % Calories from Protein | % |
| protein | Gluten | g |
| protein | Animal Protein | g |
| protein | Total Protein | g |
| protein | Vegetable Protein | g |
| PUFA | % Calories from PUFA | % |
| PUFA | Omega-3 Fatty Acids | g |
| PUFA | Omega-6 Fatty Acids | g |
| PUFA | Polyunsaturated to Saturated Fat Ratio | ? |
| PUFA | PUFA 18:2 n-6 (linoleic acid [LA]) | g |
| PUFA | PUFA 18:3 n-3 (alpha-linolenic acid [ALA]) | g |
| PUFA | PUFA 18:3 n-6 (gamma-linolenic acid [GLA]) | g |
| PUFA | PUFA 20:4 n-6 (arachidonic acid [AA]) | g |
| PUFA | PUFA 18:2 (linoleic acid, undifferentiated) | g |
| PUFA | PUFA 18:3 (linolenic acid, undifferentiated) | g |
| PUFA | PUFA 18:4 (parinaric acid) | g |
| PUFA | PUFA 20:4 (arachidonic acid, undifferentiated) | g |
| PUFA | PUFA 20:5 n-3 (eicosapentaenoic acid [EPA]) | g |
| PUFA | PUFA 22:5 n-3 (docosapentaenoic acid [DPA]) | g |
| PUFA | PUFA 22:6 n-3 (docosahexaenoic acid [DHA]) | g |
| SFA | % Calories from SFA | % |
| SFA | SFA 10:0 (capric acid) | g |
| SFA | SFA 12:0 (lauric acid) | g |
| SFA | SFA 14:0 (myristic acid) | g |
| SFA | SFA 16:0 (palmitic acid) | g |
| SFA | SFA 17:0 (margaric acid) | g |
| SFA | SFA 18:0 (stearic acid) | g |
| SFA | SFA 20:0 (arachidic acid) | g |
| SFA | SFA 22:0 (behenic acid) | g |
| SFA | SFA 4:0 (butyric acid) | g |
| SFA | SFA 6:0 (caproic acid) | g |
| SFA | SFA 8:0 (caprylic acid) | g |
| sweeteners and food additives | Maltitol | g |
| sweeteners and food additives | Aspartame | mg |
| sweeteners and food additives | Erythritol | g |
| sweeteners and food additives | Isomalt | g |
| sweeteners and food additives | Lactitol | g |
| sweeteners and food additives | Maltose | g |
| sweeteners and food additives | Saccharin | mg |
| sweeteners and food additives | Sorbitol | g |
| sweeteners and food additives | Sucralose | mg |
| sweeteners and food additives | Xylitol | g |
| total MUFA | Total Monounsaturated Fatty Acids (MUFA) | g |
| total PUFA | Total Polyunsaturated Fatty Acids (PUFA) | g |
| total SFA | Total Saturated Fatty Acids (SFA) | g |
| total trans-fat | Total Trans-Fatty Acids (TRANS) | g |
| trans-fat | 16:1 TRANS (trans-hexadecenoic acid) | g |
| trans-fat | 18:1 TRANS (trans-octadecenoic acid) | g |
| trans-fat | 18:2 TRANS (trans-octadecadienoic acid) | g |
| trans-fat | CLA cis-9, trans-11 | g |
| trans-fat | CLA trans-10, cis-12 | g |
| trans-fat | Total Conjugated Linoleic Acid (CLA 18:2) | g |
| vitamins | Vitamin D2 (ergocalciferol) | mcg |
| vitamins | Vitamin D3 (cholecalciferol) | mcg |
| vitamins | Alpha-Carotene (provitamin A carotenoid) | mcg |
| vitamins | Beta-Carotene (provitamin A carotenoid) | mcg |
| vitamins | Beta-Carotene Equivalents (derived from provitamin A carotenoids) | mcg |
| vitamins | Beta-Cryptoxanthin (provitamin A carotenoid) | mcg |
| vitamins | Beta-Tocopherol | mg |
| vitamins | Choline | mg |
| vitamins | Delta-Tocopherol | mg |
| vitamins | Dietary Folate Equivalents | mcg |
| vitamins | Gamma-Tocopherol | mg |
| vitamins | Lutein + Zeaxanthin | mcg |
| vitamins | Lycopene | mcg |
| vitamins | Natural Folate (food folate) | mcg |
| vitamins | Niacin (vitamin B3) | mg |
| vitamins | Niacin Equivalents | mg |
| vitamins | Pantothenic Acid | mg |
| vitamins | Retinol | mcg |
| vitamins | Riboflavin (vitamin B2) | mg |
| vitamins | Synthetic Alpha-Tocopherol (all rac-alpha-tocopherol or dl-alpha-tocopherol) | mg |
| vitamins | Synthetic Folate (folic acid) | mcg |
| vitamins | Thiamin (vitamin B1) | mg |
| vitamins | Total Alpha-Tocopherol Equivalents (see columns 171-173) | mg |
| vitamins | Total Folate (see columns 149-151) | mcg |
| vitamins | Total Vitamin A Activity (International Units) | IU |
| vitamins | Total Vitamin A Activity (Retinol Activity Equivalents) | mcg |
| vitamins | Total Vitamin A Activity (Retinol Equivalents) | mcg |
| vitamins | Vitamin B12 (cobalamin) | mcg |
| vitamins | Vitamin B6 (pyridoxine, pyridoxyl, and pyridoxamine) | mg |
| vitamins | Vitamin C (ascorbic acid) | mg |
| vitamins | Vitamin D (calciferol) | mcg |
| vitamins | Vitamin E (International Units) | IU |
| vitamins | Vitamin E (Total Alpha-Tocopherol) (see columns 171-173) | mg |
| vitamins | Vitamin K (phylloquinone) | mcg |
| water | Water | g |
|  |  |  |
|  |  |  |

| **Supplemental Table 3. Transcription factors predicted to bind to enhancer elements containing selected differential methylation sites.** | | | | |
| --- | --- | --- | --- | --- |
| **Transcription factor*** | **GH07J150372** | **GH20J006764** | **GH20J012950** | **GH0XJ013211** |
| AFF1 | yes | no | no | no |
| AHR | yes | no | no | no |
| ARID1B | yes | no | no | no |
| ARID3A | yes | no | no | no |
| ARID4B | yes | no | no | no |
| ARNT | yes | no | no | no |
| ATF1 | yes | no | no | no |
| ATF7 | yes | no | no | no |
| BACH1 | yes | yes | no | no |
| BCL11A | yes | no | no | no |
| BCL6 | yes | no | no | no |
| BCLAF1 | yes | no | no | no |
| BCOR | yes | no | no | no |
| BHLHE40 | yes | yes | no | no |
| BMI1 | yes | no | no | no |
| BRD9 | yes | no | no | no |
| CBFA2T3 | yes | no | no | no |
| CBX1 | yes | no | no | no |
| CBX2 | no | yes | no | no |
| CEBPZ | yes | no | no | no |
| CHD1 | yes | no | no | no |
| CHD2 | yes | no | no | no |
| CREM | yes | no | no | no |
| CTBP1 | yes | yes | no | no |
| CTCF | yes | yes | no | no |
| DACH1 | yes | no | no | no |
| DDX20 | yes | no | no | no |
| DMAP1 | yes | no | no | no |
| DNMT3B | yes | no | no | no |
| DPF2 | yes | yes | no | no |
| DRAP1 | yes | no | no | no |
| EBF1 | yes | no | no | no |
| EGR2 | yes | no | no | no |
| ELF1 | yes | no | no | no |
| ELF3 | yes | no | no | no |
| EP300 | yes | no | no | yes |
| EP400 | yes | no | no | no |
| ERF | yes | no | no | no |
| ETS1 | yes | no | no | no |
| ETV6 | yes | no | no | no |
| EZH2 | no | yes | no | yes |
| FOS | yes | no | no | no |
| FOSL2 | yes | no | no | no |
| FOXA1 | yes | no | no | no |
| FOXA2 | yes | no | no | no |
| FOXA3 | yes | no | no | no |
| FOXK2 | yes | no | no | no |
| FOXP1 | yes | no | no | no |
| GATA4 | yes | no | no | no |
| GATAD1 | yes | no | no | no |
| GATAD2A | yes | no | no | no |
| GATAD2B | yes | yes | no | no |
| GLI4 | yes | no | no | no |
| GLIS1 | yes | no | no | no |
| GLIS2 | yes | no | no | no |
| GMEB1 | yes | no | no | no |
| GTF2A2 | yes | no | no | no |
| GTF2F1 | yes | no | no | no |
| HDAC1 | yes | no | no | no |
| HDAC2 | yes | yes | no | no |
| HDGF | yes | yes | no | no |
| HIC1 | yes | yes | no | no |
| HLF | yes | yes | no | no |
| HMBOX1 | yes | no | no | no |
| HNF1A | yes | no | no | no |
| HNF4A | yes | yes | no | no |
| HNRNPH1 | yes | no | no | no |
| HNRNPL | yes | no | no | no |
| IKZF1 | yes | yes | no | no |
| IKZF2 | yes | yes | no | no |
| IKZF5 | yes | no | no | no |
| IRF2 | yes | no | no | no |
| IRF3 | yes | no | no | no |
| IRF4 | yes | no | no | no |
| JUND | no | no | no | yes |
| KAT7 | yes | no | no | no |
| KAT8 | yes | no | no | no |
| KDM1A | yes | no | no | no |
| KDM3A | yes | no | no | no |
| KDM6A | yes | no | no | no |
| KLF1 | yes | no | no | no |
| KLF10 | yes | no | no | no |
| KLF11 | yes | no | no | no |
| KLF5 | yes | no | no | no |
| KLF7 | yes | no | no | no |
| KMT2B | yes | no | no | no |
| L3MBTL2 | yes | no | no | no |
| LARP7 | yes | yes | no | no |
| MAX | yes | no | no | no |
| MAZ | yes | no | no | no |
| MBD1 | yes | no | no | no |
| MBD2 | yes | no | no | no |
| MEIS2 | yes | no | no | no |
| MGA | yes | no | no | no |
| MIER1 | yes | no | no | no |
| MIER3 | yes | no | no | no |
| MIXL1 | yes | no | no | no |
| MLLT1 | yes | no | no | no |
| MNT | yes | no | no | no |
| MTA1 | no | yes | no | no |
| MTA2 | yes | no | no | no |
| MTA3 | yes | no | no | no |
| MXD3 | yes | no | no | no |
| MYC | yes | no | no | no |
| MYNN | yes | no | no | no |
| MZF1 | yes | no | no | no |
| NANOG | yes | yes | no | no |
| NBN | yes | yes | no | no |
| NCOR1 | yes | no | no | no |
| NFATC1 | yes | no | no | no |
| NFIB | yes | yes | no | no |
| NFIC | yes | no | no | no |
| NFKBIZ | yes | no | no | no |
| NFRKB | yes | no | no | no |
| NFYC | yes | no | no | no |
| NKRF | yes | yes | no | no |
| NONO | yes | no | no | no |
| NR2C1 | yes | no | no | no |
| NR2F1 | yes | yes | no | no |
| NR2F2 | yes | no | no | no |
| NR2F6 | yes | no | no | no |
| NRF1 | yes | no | no | no |
| NUFIP1 | yes | no | no | no |
| OSR2 | yes | no | no | no |
| PBX2 | yes | no | no | no |
| PHF20 | yes | no | no | no |
| PHF21A | yes | no | no | no |
| PHF8 | yes | no | no | no |
| PKNOX1 | yes | yes | no | no |
| PML | yes | no | no | no |
| POLR2A | yes | yes | no | no |
| POLR2B | yes | no | no | no |
| POU5F1 | yes | no | no | no |
| PPARG | yes | no | no | no |
| PRDM10 | no | yes | no | no |
| PRDM2 | yes | no | no | no |
| PRDM4 | yes | no | no | no |
| RAD21 | yes | yes | no | no |
| RAD51 | yes | no | no | no |
| RARA | yes | no | no | no |
| RB1 | yes | yes | no | no |
| RBFOX2 | yes | no | no | no |
| RBM22 | yes | no | no | no |
| RBPJ | yes | no | no | no |
| RCOR1 | yes | no | no | no |
| RELB | yes | yes | no | no |
| REPIN1 | yes | no | no | no |
| RERE | yes | no | no | no |
| REST | yes | no | no | no |
| RFX1 | yes | no | no | no |
| RFX3 | yes | no | no | no |
| RFX5 | yes | no | no | no |
| RNF2 | yes | yes | no | no |
| RUNX1 | yes | no | no | no |
| RUNX3 | yes | no | no | no |
| RXRA | yes | no | no | no |
| RXRB | yes | no | no | no |
| SAP130 | yes | no | no | no |
| SIN3A | yes | yes | no | no |
| SIN3B | yes | no | no | no |
| SKI | yes | yes | no | no |
| SKIL | yes | no | no | no |
| SMAD4 | yes | no | no | no |
| SMAD5 | yes | no | no | no |
| SMARCA4 | yes | no | no | no |
| SMARCA5 | yes | yes | no | no |
| SMARCE1 | yes | no | no | no |
| SMC3 | yes | yes | no | no |
| SOX13 | yes | no | no | no |
| SOX5 | yes | no | no | no |
| SOX6 | yes | no | no | no |
| SP1 | yes | no | no | no |
| SP2 | yes | no | no | no |
| SP3 | yes | no | no | no |
| SP5 | yes | no | no | no |
| SP7 | yes | no | no | no |
| SPI1 | yes | no | no | no |
| SREBF1 | yes | no | no | no |
| STAT5A | yes | no | no | no |
| SUPT5H | yes | no | no | no |
| TAF1 | yes | no | no | no |
| TAF7 | yes | no | no | no |
| TAL1 | yes | no | no | no |
| TARDBP | yes | no | no | no |
| TBL1XR1 | yes | no | no | no |
| TBP | yes | no | no | no |
| TCF12 | yes | no | no | no |
| TCF7 | yes | no | no | no |
| TCF7L2 | yes | yes | no | no |
| TEAD1 | yes | no | no | no |
| TEAD3 | yes | no | no | no |
| TFAP4 | yes | yes | no | no |
| TGIF2 | yes | no | no | no |
| THAP11 | yes | no | no | no |
| TRIM22 | yes | yes | no | no |
| TRIM25 | yes | no | no | no |
| TRIM28 | yes | no | no | no |
| U2AF1 | yes | no | no | no |
| U2AF2 | yes | no | no | no |
| UBTF | yes | no | no | no |
| USF1 | yes | yes | no | no |
| USF2 | yes | no | no | no |
| XRCC5 | yes | no | no | no |
| YY1 | yes | yes | no | no |
| YY2 | yes | no | no | no |
| ZBED1 | yes | no | no | no |
| ZBTB10 | yes | no | no | no |
| ZBTB17 | yes | yes | no | no |
| ZBTB2 | yes | no | no | no |
| ZBTB20 | yes | yes | no | no |
| ZBTB21 | yes | no | no | no |
| ZBTB25 | yes | no | no | no |
| ZBTB26 | yes | yes | no | no |
| ZBTB33 | yes | no | no | no |
| ZBTB40 | yes | no | no | no |
| ZBTB48 | yes | no | no | no |
| ZBTB6 | yes | no | no | no |
| ZBTB7A | yes | no | no | no |
| ZBTB8A | yes | no | no | no |
| ZEB2 | yes | no | no | no |
| ZFHX2 | yes | no | no | no |
| ZFP36 | no | yes | no | no |
| ZFP37 | yes | no | no | no |
| ZFP64 | no | yes | no | no |
| ZFP69B | yes | no | no | no |
| ZFP91 | yes | no | no | no |
| ZFX | yes | no | no | no |
| ZGPAT | yes | no | no | no |
| ZHX2 | yes | no | no | no |
| ZIC2 | yes | no | no | no |
| ZKSCAN1 | yes | no | no | no |
| ZNF10 | yes | no | no | no |
| ZNF121 | yes | no | no | no |
| ZNF140 | yes | no | no | no |
| ZNF184 | yes | no | no | no |
| ZNF189 | yes | no | no | no |
| ZNF205 | yes | no | no | no |
| ZNF207 | yes | no | no | no |
| ZNF217 | yes | yes | no | no |
| ZNF24 | yes | yes | no | no |
| ZNF280C | yes | no | no | no |
| ZNF316 | yes | no | no | no |
| ZNF335 | yes | yes | no | no |
| ZNF341 | yes | yes | no | no |
| ZNF366 | yes | no | no | no |
| ZNF384 | no | yes | no | no |
| ZNF398 | yes | no | no | no |
| ZNF423 | yes | no | no | no |
| ZNF48 | yes | no | no | no |
| ZNF501 | yes | no | no | no |
| ZNF511 | yes | no | no | no |
| ZNF561 | yes | no | no | no |
| ZNF580 | yes | no | no | no |
| ZNF592 | yes | no | no | no |
| ZNF600 | yes | no | no | no |
| ZNF610 | yes | no | no | no |
| ZNF623 | yes | no | no | no |
| ZNF644 | yes | no | no | no |
| ZNF654 | no | yes | no | no |
| ZNF664 | yes | no | no | no |
| ZNF687 | yes | no | no | no |
| ZNF692 | yes | no | no | no |
| ZNF843 | yes | no | no | no |
| ZSCAN16 | yes | no | no | no |
| ZSCAN23 | yes | no | no | no |
| ZSCAN29 | yes | yes | no | no |
| ZSCAN9 | yes | no | no | no |
| ZXDB | yes | no | no | no |
| *Predictions of transcription factor binding are taken from reference 41 | | | |  |
|  |  |  |  |  |
